# Tomato leaf curl New Delhi virus: an emerging plant begomovirus threatening cucurbit production

**DOI:** 10.1007/s42994-023-00118-4

**Published:** 2023-10-25

**Authors:** Lingmin Cai, Yuzhen Mei, Ruyi Ye, Yun Deng, Xuejun Zhang, Zhongyuan Hu, Xueping Zhou, Mingfang Zhang, Jinghua Yang

**Affiliations:** 1 Laboratory of Germplasm Innovation and Molecular Breeding, Institute of Vegetable Science, Zhejiang University, Hangzhou, 310058 China; 2 Institute of Biotechnology, Zhejiang University, Hangzhou, 310058 China; 3 Peking University Institute of Advanced Agricultural Sciences, Weifang, 261000 China; 4 Hami Melon Research Center, Xinjiang Academy of Agricultural Sciences, Urumqi, 830091 China; 5 Hainan Institute, Zhejiang University, Yazhou Bay Science and Technology City, Sanya, 572025 China; 6 Key Laboratory of Horticultural Plant Growth and Development, Ministry of Agriculture and Rural Affairs, Hangzhou, 310058 China

**Keywords:** Tomato leaf curl New Delhi virus, Cucurbits, Emerging virus disease, Genetic resistance

## Abstract

Tomato leaf curl New Delhi virus (ToLCNDV), a bipartite begomovirus, was first reported to infect tomato and has recently spread rapidly as an emerging disease to *Cucurbitaceae* crops. To date, the virus has been reported to infect more than 11 cucurbit crops, in 16 countries and regions, causing severe yield losses. In autumn 2022, ToLCNDV was first isolated from cucurbit plants in Southeastern coastal areas of China. Phylogenetic analysis established that these isolates belong to the Asian ToLCNDV clade, and shared high nucleotide identity and closest genetic relationship with the DNA-A sequence from the Chinese tomato-infecting ToLCNDV isolate (Accession no. OP356207) and the tomato New Delhi ToLCNDV-Severe isolate (Accession no. HM159454). In this review, we summarize the occurrence and distribution, host range, detection and diagnosis, control strategies, and genetic resistance of ToLCNDV in the *Cucurbitaceae*. We then summarize pathways that could be undertaken to improve our understanding of this emerging disease, with the objective to develop ToLCNDV-resistant cucurbit cultivars*.*

**Supplementary Information:**

The online version contains supplementary material available at 10.1007/s42994-023-00118-4.

## Introduction

Begomoviruses are a serious threat to many economically important crops and cause devastating diseases, worldwide (Navas-Castillo et al. 2011). Based on their genome characteristics, host range, and insect vector, the family Geminiviridae is currently divided into fourteen genera, with Begomovirus being the largest genus (Brown et al. 2015; Varsani et al. 2014). Begomoviruses are classified as monopartite (with a single genomic component) or bipartite (with genomic components referred to as DNA A and DNA B). These geminiviruses are transmitted by the whitefly, *Bemisia tabaci* (Gennadius) (order: Hemiptera, family: Aleyrodidae), in a circulatory persistent manner and infect many dicotyledonous plant species (Brown et al. 2015).

Tomato leaf curl New Delhi virus (ToLCNDV) was first described in 1995 infecting tomato (*Solanum lycopersicum*) in northern India. This virus is a rapidly spreading bipartite begomovirus, with two circular single-stranded DNA genomic components each of approx. 2.7 kb, designated as DNA-A and DNA-B (Srivastava et al. 1995). ToLCNDV is transmitted by whiteflies but can also be inoculated mechanically (Lopez et al. 2015; Tsai et al. 2011). Typical symptoms include severe leaf curling, yellow mosaic patterns and swelling of the veins of young leaves and dwarfing through the shortening of internodes (Zaidi et al. 2017).

Following its first report 28 years ago, ToLCNDV has been reported to infect a large number of economically important crops, over a wide range of families, including *Cucurbitaceae*, *Solanaceae*, *Malvaceae*, *Fabaceae* and *Euphorbiaceae* (Seal et al. 2006). Particularly in the cucurbits, ToLCNDV can cause severe losses in such vegetables as zucchini (*Cucurbita pepo*), cucumber (*Cucumis sativus*), and squash (*Cucurbita moschata*), and in fruits such as melon (*Cucumis melo*) and watermelon (*Citrullus lanatus*), as well as several pumpkin species (Bandaranayake et al. 2014; Siskos et al. 2022; Venkataravanappa et al. 2020; Yamamoto et al. 2021). In Europe, ToLCNDV has had severe negative impacts on various crops, especially cucurbits, with losses of up to 20% in open-field melon production in central Spain (Saez et al. 2017).

Genetic resistance has been identified in several *Cucurbitaceae* crops, including melon, cucumber, pumpkin, and sponge gourd (*Luffa cylindrica*) (Kaur et al. 2021; Romero-Masegosa et al. 2021; Saez et al. 2017, 2020, 2021). However, to date, no resistance gene has been successfully mapped and characterized.

In the autumn of 2022, ToLCNDV was isolated from different cucurbit crops being grown in Jiangsu Province, Zhejiang Province and Shanghai, located in the Southeastern coastal areas of China, where infection of ~ 650 hectares caused approx. $US15 million in economic losses (Gu et al. 2023; Zeng et al. 2023).

Here, we briefly describe the history of ToLCNDV research in the *Cucurbitaceae* and then address the distribution, host range, genetic relationship, detection and diagnosis, control strategies, and genetic resistance of ToLCNDV. We then assess the presence of natural sources of resistant germplasm and mapping of resistance genes, which could be employed to advance our knowledge on this emerging disease in the cucurbits and other crops.

### Discovery and distribution

Although ToLCNDV was first described in India in 1995, only partial DNA fragments of begomoviruses were detected from cucurbits in 2000, including cucumber, muskmelon (*Cucumis melo L.*), cantaloupe melon (*Cucumis melo var. reliculatus*) and wax gourd (*Benincase hispida*) (Samretwanich et al. 2000a, 2000b, 2000c). Subsequently, several disease reports indicated that ToLCNDV had caused severe symptoms in bitter gourd, in Pakistan (Tahir and Haider 2005), and infected cucumber, bottle gourd and muskmelon, in Thailand (Ito et al. 2008). The distribution of ToLCNDV in the cucurbits has now been reported in the Indian subcontinent, Southeast Asia, and East Asia. Since 2012, reports of ToLCNDV-infected cucurbits have spread westward, including from Algeria, Spain, Morocco, Tunisia, Italy, Greece, and Iran (Juarez et al. 2014; Kheireddine et al. 2019; Mnari-Hattab et al. 2015; Orfanidou et al. 2019; Panno et al. 2016; Sifres et al. 2018; Yazdani-Khameneh et al. 2016). In recent years, additional countries (Malaysia, Cambodia, Laos, Indonesia, and France) have reported that ToLCNDV has caused problems in cucurbit crops (Chen et al. 2021; Desbiez et al. 2021; Neoh et al. 2023).

To date, according to the retrieved data from NCBI, more than 11 infected cucurbit species have been reported from 16 countries and areas in three continents, including Asia, Europe, and Africa (Table 1). These regions are located mainly in tropical and temperate regions, where ToLCNDV has caused tremendous economic losses in both greenhouse and open-field production of cucurbit crops (Lopez et al. 2015).

**Table 1:** Lists of reporting ToLCNDV in *Cucurbit* crops across the globe

Country	*Cucurbit* species	Scientific name	Year	Accession number	No. of accession
Algeria	Cucumber	*Cucumis sativus*	2019	MK981891	1
Cambodia	Cucumber	*Cucumis sativus*	2020	MT682358	1
	Ridge gourd	*Luffa acutangula*	2019	MN630276	9
	Wax gourd	*Benincase hispida*	2018	MH328257	1
France	Zucchini	*Cucurbita pepo*	2020	MW310624	1
India	Ash gourd	*Benincasa hispida*	2011	JN208136	2
	Bitter gourd	*Momordica charantia*	2015	KP868764	6
	Cucumber	*Cucumis sativus*	2013	KC545812	2
	Ivy gourd	*Coccinia grandis*	2017	KY780201	2
	Pumpkin	*Cucurbita moschata*	2006	AM286433	3
	Ridge gourd	*Luffa acutangula*	2015	KT426903	6
	Sponge gourd	*Luffa cylindrica*	2005	AY939926	5
	Watermelon	*Citrullus lanatus*	2018	MK087116	1
Indonesia	Cucumber	*Cucumis sativus*	2019	LC511775	1
Iran	Cucumber	*Cucumis sativus*	2015	KP641675	1
	Melon	*Cucumis melo*	2015	KP641673	2
Italy	Zucchini	*Cucurbita pepo*	2019	MK756107	1
Laos	Sponge gourd	*Luffa cylindrica*	2018	MH328254	1
Malaysia	Bitter gourd	*Momordica charantia*	2020	MW248653	3
	Cucumber	*Cucumis sativus*	2020	MW248641	10
	Melon	*Cucumis melo*	2020	MT912475	1
	Ridge gourd	*Luffa acutangula*	2020	MW248639	3
	Squash	*Cucurbita pepo*	2020	MW248655	1
	Wax gourd	*Benincase hispida*	2020	MW248643	3
Morocco	Zucchini	*Cucurbita pepo*	2017	MG098230	1
Pakistan	Cucumber	*Cucumis sativus*	2021	OM102555	5
	Sponge gourd	*Luffa cylindrica*	2017	KY933708	2
	Zucchini	*Cucurbita pepo*	2015	KT948072	1
	Zucchini	*Cucurbita pepo*	2020	MT800825	4
Spain	Cucumber	*Cucumis sativus*	2020	LC596380	1
	Cucumber	*Cucumis sativus*	2018	MH577696	7
	Melon	*Cucumis melo*	2020	LC596381	1
	Melon	*Cucumis melo*	2018	MH577702	23
	Pumpkin	*Cucurbita moschata*	2018	MH577694	6
	Zucchini	*Cucurbita pepo*	2013	KF749223	2
	Zucchini	*Cucurbita pepo*	2018	MH577683	39
	Zucchini	*Cucurbita pepo*	2020	LC596382	1
Tunisia	Cucumber	*Cucumis sativus*	2017	MF967014	3
	Melon	*Cucumis melo*	2017	MF967015	2
	Pumpkin	*Cucurbita moschata*	2017	MF967019	3
China	Melon	*Cucumis melo*	2022	OR157979	14
Greece	Zucchini	*Cucurbita pepo*	2019	KM977733	1
Thailand	Cucumber	*Cucumis sativus*	2007	AB330079	1
	Bottle gourd	*Lagenaria siceraria*	2007	AB368447	1
	Melon	*Cucumis melo*	2007	AB368448	1

Data is retrieved from NCBI

### Host range and disease symptoms in *Cucurbits*

ToLCNDV infects a wide range of hosts, which in the cucurbits include the following: melon, cucumber, watermelon, zucchini, pumpkin, gourd, sponge gourd, wax gourd, bottle gourd (*Lagenaria siceraria*), ridge gourd (*Luffa acutangula*), bitter gourd (*Momordica charantia*), ivy gourd (*Coccinia grandis*), ash gourd (Bragard et al. 2020) (Table 1). Symptoms consist of severe yellowing and mosaic discoloration in young leaves and includes leaf curling, vein swelling and short internodes, along with fruit skin roughness and longitudinal cracking (Bragard et al. 2020; Juarez et al. 2019; Lopez et al. 2015).

**Fig. 1:**
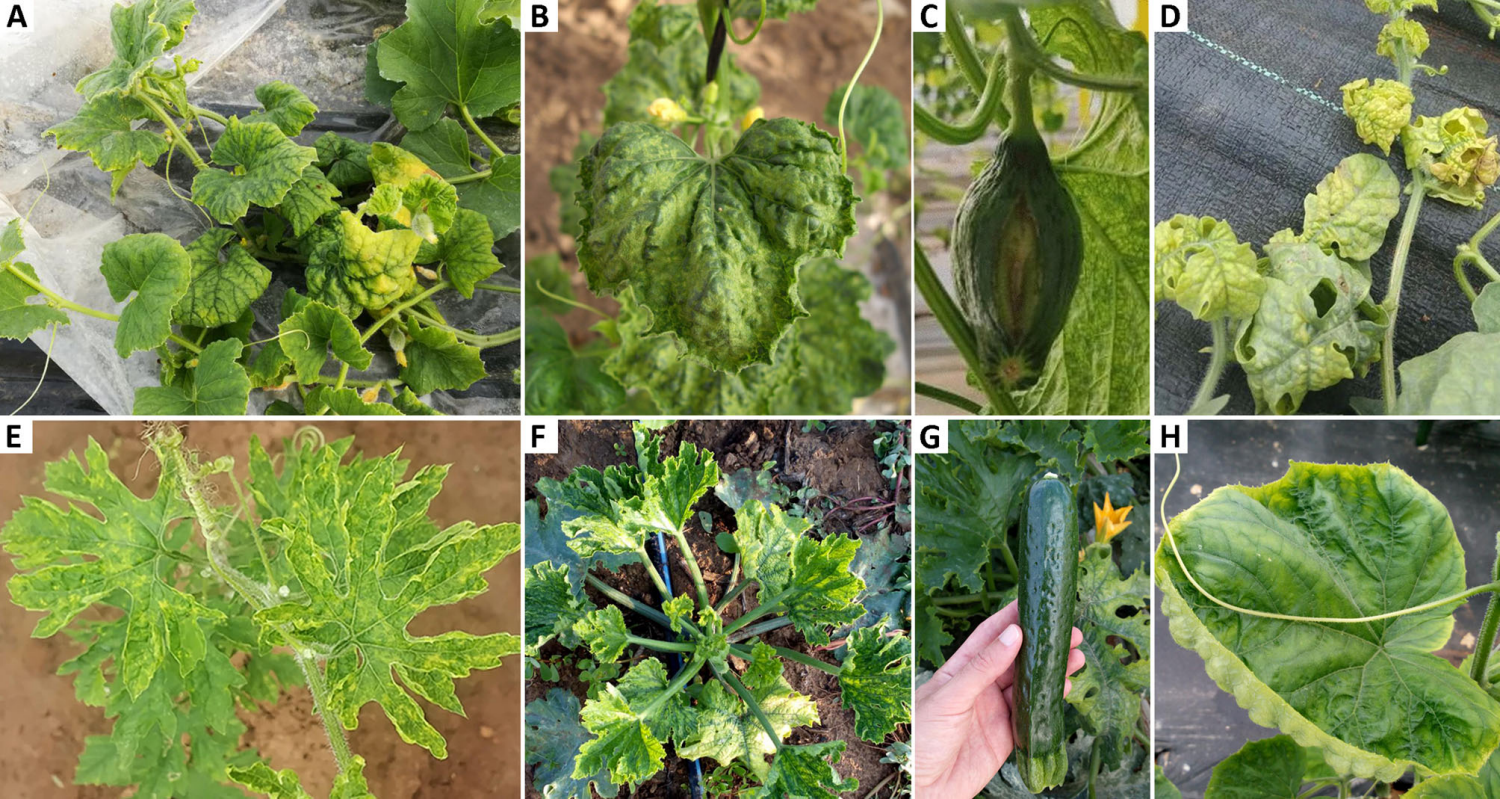
Disease symptoms caused by Tomato leaf curl New Delhi virus in foliage and fruit of different cucurbit species. Yellowing and curling of melon (*Cucumis melo*) leaves (**A**, **B**), as well as fruit cracking (**C**). Watermelon (*Citrullus lanatus*) plants show yellowing and downward curling symptoms (**D**). Mosaic mottling with slight curling of leaves in bitter gourd (*Lagenaria siceraria*) (**E**). Short internodes and curling, vein swelling and mosaic symptoms in young zucchini (*Cucurbita pepo*) leaves (**F**). Zucchini fruits with lower marketability due to the roughness of the skin and reduced size (**G**). Yellowing and upward leaf curling on cucumber (*Cucumis sativus*) leaves (**H**)**.** Images in (**A**), (**F**), (**G**), and (**H**) refer to the European and Mediterranean Plant Protection Organization (EPPO) global database (https://gd.eppo.int/taxon/TOLCND/photos), posted by Dr. Raffaele Giurato. Figures 1B, C were cited from Siskos et al. (2022) without revision; reprint permission: https://link.springer.com/article/10.1007/s10681-022-03081-1#rightslink; Fig. 1D was cited from Venkataravanappa et al. (2020) without revision, reprint permission: https://s100.copyright.com/CustomerAdmin/PLF.jsp?ref=e2f56b44-78b5-4777-b376-7ab99eb6039e; Fig. 1E was cited from Kiran et al. (2021) without revision; reprint permission: https://s100.copyright.com/CustomerAdmin/PLF.jsp?ref=625141e1-4cce-4fc2-90dc-f95c540cdfe8. Data is retrieved from NCBI

Short internodes and curling, vein swelling and mosaic in young leaves were observed on the leaves of melon (Fig. 1A, B) and melon fruits have longitudinal cracks (Fig. 1C) (Siskos et al. 2022). Infected bitter gourd shows mosaic mottling with slight curling of leaves (Fig. 1E) (Kiran et al. 2021). The disease leaves symptoms of zucchini were similar to melon (Fig. 1F) (Juarez et al. 2014) and fruits are of lower quality due to skin roughness and reduced size (Fig. 1G). Watermelon and cucumber leaves show yellowing and upward leaf curling (Fig. 1D, H) (Venkataravanappa et al. 2020; Yamamoto et al. 2021).

## Genome characterization and sequence diversity

ToLCNDV is a bipartite begomovirus with two circular single-stranded DNA genome components: DNA-A contains the AV1 and AV2 genes, in the virion sense orientation, which encode the coat protein (CP) and pre-coat protein, respectively. In the complementary sense orientation, AC1, AC2, AC3 and AC4 encode the replication-associated protein (Rep), transcriptional activator protein (TrAP), replication enhancer protein (REn) and viral effector, respectively. ToLCNDV DNA-B consists of the BV1 gene, in the virion sense orientation, and the BC1 gene, in the complementary sense orientation, which functions as a nuclear shuttle protein (NSP) and movement protein (MP), respectively. Beta satellites are associated with ToLCNDV (Zaidi et al. 2017). ToLCNDV is unique in that it shares its DNA-B component with several other bipartite begomoviruses, such as Pepper leaf curl Bangladesh virus, Tomato leaf curl Palampur virus, and Bhendi yellow vein mosaic virus (Zaidi et al. 2016).

**Fig. 2:**
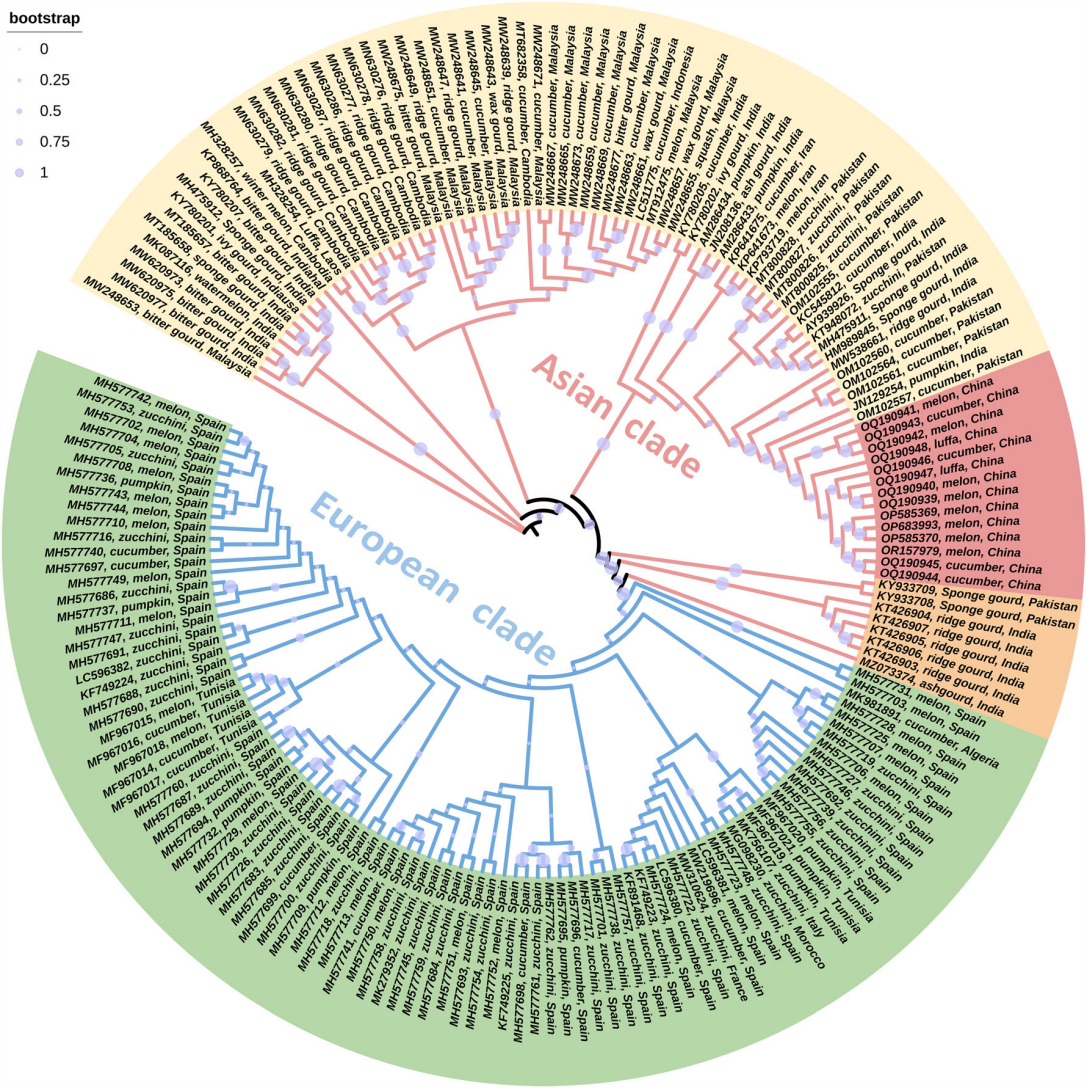
Phylogenetic trees derived from the DNA-A components of Tomato leaf curl New Delhi virus isolates derived from cucurbit plants. The genetic relationships indicate that clade separation was likely not associated with the species, but rather with the various regions. Scale bar representing the genetic distance is shown in the upper left

Globally, some 681 ToLCNDV DNA-A accessions have been deposited in the GenBank database, of which 184 are for the cucurbits (Supplemental Table 1 and 2). A phylogenetic analysis of ToLCNDV isolates from cucurbit plants indicated the presence of two main clades, an Asian and European clade, respectively (Fig. 2). The genetic relationship between these ToLCNDV cucurbit isolates is not associated with the species, but probably relates to the region.

## Detection and diagnosis

ToLCNDV serves as an emerging threat to the global cucurbit industry, and rapid and accurate detection and diagnosis are critical for an effective response to control its spread. Several molecular approaches are available for the detection and identification of ToLCNDV. Polymerase chain reaction (PCR), rolling circle amplification (RCA), sequence analysis and enzyme-linked immunosorbent assay (ELISA) methods can be routinely used for virus detection. The procedures for viral genome sequencing are as follows: Collect diseased samples from cucurbit plants showing symptoms of mosaic, leaf curl; viral DNA is then extracted from approx. 50 mg of dried samples (Gilbertson et al. 1991; Tsai et al. 2011), then specific primers are used for amplification (Chen et al. 2021; Tsai et al. 2011), and the products are sequenced, by next-generation sequencing (NGS), which provides an effective method for virus detection and identification. NGS has been successfully used to sequence the full-length ToLCNDV genome, which has offered a foundation for developing an understanding of evolutionary relationships and the global distribution of ToLCNDV (Chen et al. 2021; Figas et al. 2017).

Commercial ELISA tests (e.g., AGDIA, DSMZ) are available for ToLCNDV detection, and a molecular test, based on loop-mediated isothermal amplification (LAMP)-based molecular test, is available from Enbiotech srl (Palermo, Italy), which affords robust virus detection (Bragard et al. 2020; Panno et al. 2019). The alpha and beta satellites that are associated with ToLCNDV disease can also be detected by molecular means using appropriate primers (quantitative PCR and RCA) (Zaidi et al. 2016).

## Emergence of ToLCNDV infecting cucurbits in China

**Fig. 3:**
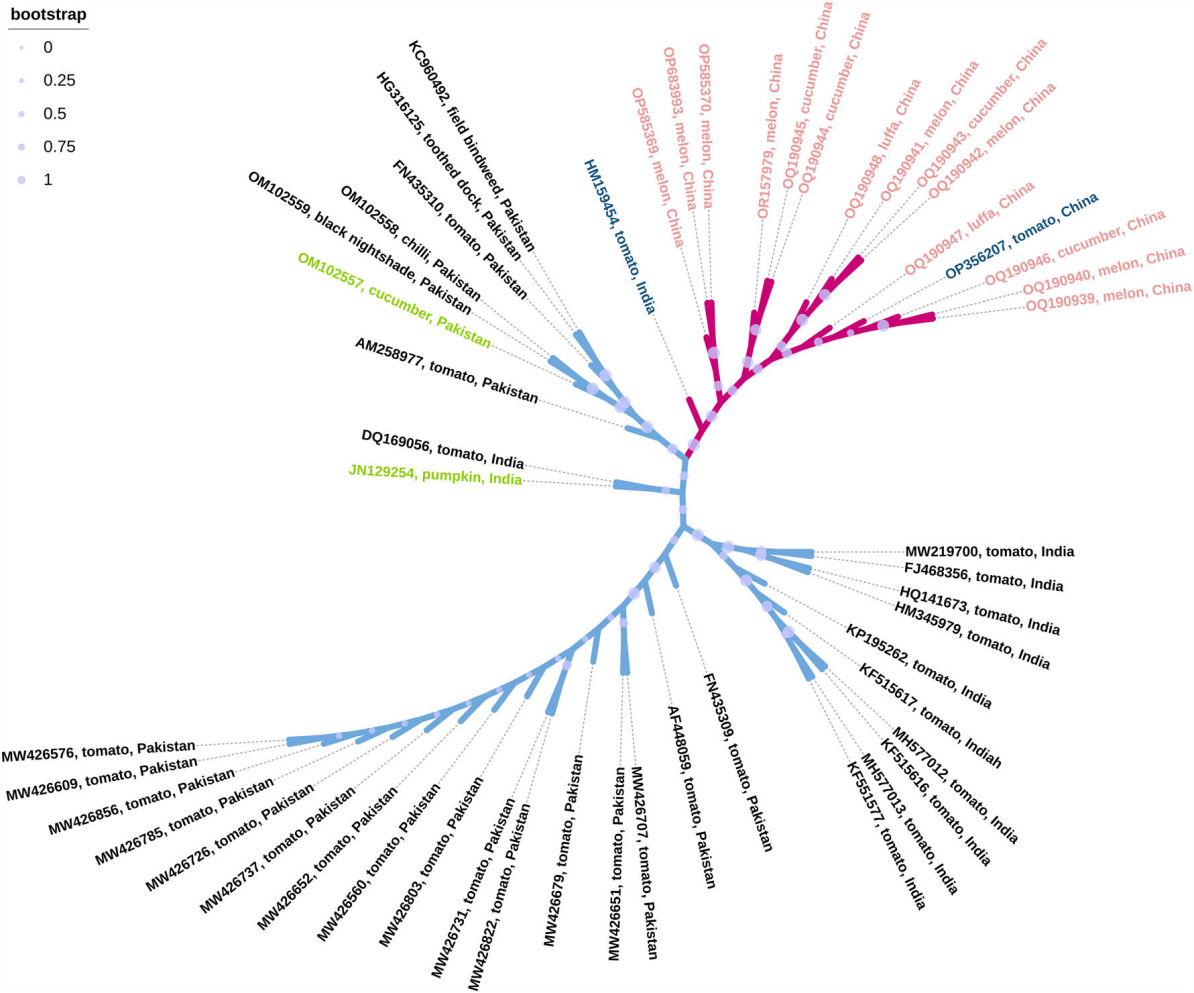
Phylogenetic trees of the DNA-A component of ToLCNDV China isolates with other selected ToLCNDV identified from different crops. The ToLCNDV China isolates from cucurbit plants are labeled in pink, and two isolates from tomato plants genetically close to the cucurbits isolates are labeled in blue, two other cucurbit isolates from India and Pakistan are labeled in green. Scale bar representing the genetic distance is shown in the upper left

In the autumn of 2022, the occurrence in China of ToLCNDV-infected cucurbit plants was reported in four cities. A phylogenetic tree, constructed with 681 ToLCNDV DNA-A accessions, and 50 isolates, most closely related to the ToLCNDV China isolates from cucurbit plants, were used for an evolutionary analysis. All the DNA-A component of ToLCNDV China cucurbit plants isolates shared high nucleotide identities (99.34–99.85%) with the DNA-A sequence of isolates of ToLCNDV infecting tomato in China (OP356207) (Li et al. 2022) (Fig. 3), and shared 98.36–98.83% with DNA-A genome of ToLCNDV-Severe isolate (Accession no. HM159454) from tomato in New Delhi. This evolutionary analysis suggests that the isolates from cucurbit plants in China were more closely related to the tomato isolates than to other cucurbit isolates from other geographical regions.

## Disease management

Integrated disease management (IDM) strategies, which combine host resistance, chemical, biological, and cultural control measures, can maximize viral disease control, while also meeting the requirements of environmental and social responsibility (Jones 2004). There is a consensus that effective control of ToLCNDV should be based on such an integrated management approach.

### Chemical control

*Bemisia tabaci* is a very efficient vector of ToLCNDV, and the use of insecticides has long been the main strategy for whitefly management (Legg et al. 2014). However, heavy pesticide use can lead to the emergence of resistant whitefly populations and, long term, can be ecologically harmful. Natural plant bio-stimulants can be used as environmentally friendly alternatives to agricultural chemicals. A recent study reported that such bio-stimulants (e.g., Fullcrhum Alert and BioVeg 500) affected the fitness of zucchini plants against ToCLNDV (Donati et al. 2022). Essential oils (EOs) and hydrosols (HS) derived from natural plant products have also gained attention. For example, EOs and HS from *Origanum vulgare* (OV), *Thymus vulgaris* (TV), and *Rosmarinum officinalis* (RO) were beneficial in treating ToLCNDV-infected greenhouse-grown *Cucurbita pepo* plants (Taglienti et al. 2022).

### Biological control

Biological control of insect vectors would also assist in limiting the spread of ToLCNDV. For example, the predatory mite, *Amblyseius swirskii*, which feeds on *Bemisia tabaci* eggs and nymphs (the first instar stage, also called crawlers), is an effective biological control agent. These mites can significantly reduce the secondary transmission of ToLCNDV in zucchini crops (Tellez et al. 2017). Based on these findings, a combination of pesticides compatible with predatory mites could reduce ToLCNDV primary transmission, by eliminating the *B. tabaci* adults, thus more effectively controlling ToLCNDV transmission (Rodriguez et al. 2019).

### Cultural practices

The use of healthy planting material is an important first step in minimizing the spread of ToLCNDV. As seed transmission of ToLCNDV has been reported in India and Italy (Kil et al. 2020; Sangeetha et al. 2018), strict quarantine measures are essential when cucurbit seeds are transported between production areas. Insect and virus-free material is recommended for cucurbits, such as sponge gourd, which are usually vegetatively propagated (Venkataravanappa et al. 2019). In addition, some cultural practices, such as the implementation of a fallow period, early or late planting, physical barriers, intercropping, etc., can also be used to control the whitefly population and associated viral diseases (Hilje et al. 2001). Crop selection is also an important part of disease management. As an example, bitter gourd planted adjacent to tomato plots showed susceptibility to ToLCNDV (Kiran et al. 2021). Weeds can also act as alternative viral hosts; hence, effective and timely application of weed control, is essential. Weeds reported to serve as alternative hosts for ToLCNDV include *Ecballium elaterium*, *Datura stramonium*, *Sonchus oleraceus*, and S*olanum nigrum* (Juarez et al. 2019).

### Host resistance

Resistance to ToLCNDV has been reported in several cucurbit accessions, with five resistant lines, DSG-6, DSG-7, IIHR-137, IIHR-138 and IIHR-Sel-1, identified in sponge gourd (Islam et al. 2010; Kaur et al. 2021). The DSG-6 and DSG-7 resistance to ToLCNDV was shown to be controlled by a single dominant gene (Islam et al. 2010). Three sources of resistance have been identified in squash, including the improved cultivar Large Cheese from the USA (PI 604506), an Indian landrace (PI 381814) and a Japanese accession, BSUAL-252. Here, PI 604506 resistance is conferred by a single recessive gene, located between 799,373 and 986,936 bp on Chromosome 8, whereas the BSUAL-252 resistance was shown to be controlled by a single dominant gene unrelated to this region on Chromosome 8 (Romero-Masegosa et al. 2021; Saez et al. 2016, 2020).

In melon, nine accessions with good resistance to ToLCNDV have been identified: Kharbuja, PI 124112, PI 414723, WM9, WM7, AM 87, IC-274014, PI 282448 and PI 179901 (Lopez et al. 2015; Roman et al. 2019; Saez et al. 2017). Three genomic regions, derived from the wild Indian source, WM7, were identified as resistance-conferring loci, including a major QTL on Chromosome 11 and two additional regions on Chromosomes 12 and 2 (Saez et al. 2017). Further candidate gene validation established that transcript levels of *CmARP4* and *CmNAC* were differentially higher in the inoculated susceptible genotype than in the inoculated resistant genotype (Roman et al. 2019). Studies on the inheritance of resistance in IC-274014 revealed the involvement of one recessive (*bgm-1*) and two dominant (*Bgm-2*, *Tolcndv*) genes (Romay et al. 2019).

Transcriptome analysis has also been employed to study the interactions between ToLCNDV and melon. Here, the structural functionality of differentially expressed genes (DEGs) associated with the main QTLs for ToLCNDV resistance has been reported (Saez et al. 2022). In cucumber, three resistant lines were identified, CGN23089, CGN23423, and CGN23633, in which resistance was controlled by a single recessive gene, and a resistance-related QTL was identified on Chromosome 2 (Saez et al. 2021).

## Conclusions and perspective

Cucurbit crops suffer from numerous viral diseases, with ToLCNDV being highly transmitted by whitefly vectors, and it can also be co-infected with multiple other begomoviruses (Zaidi et al. 2016). Here, we outlined the discovery of ToLCNDV in China, its global distribution, host range, detection, and diagnosis. Development of control strategies, including IDM, in combination with breeding for robust genetic resistance, are essential for the detection and control of this emerging disease in cucurbit crops.

Although progress has been accomplished, it is imperative that candidate resistance/susceptibility genes be further studied to develop an understanding of the molecular mechanism(s) by which the ToLCNDV infection process is either prevented or permitted in the host plant. The utility of such validated resistance genes would be in their development as molecular makers for use in breeding ToLCNDV-resistant varieties.

### Supplementary Information

Below is the link to the electronic supplementary material.

Supplementary file1 (XLSX 19 KB)

## Data Availability

Data sharing is not applicable to this article as no datasets were generated or analyzed during the current study.
